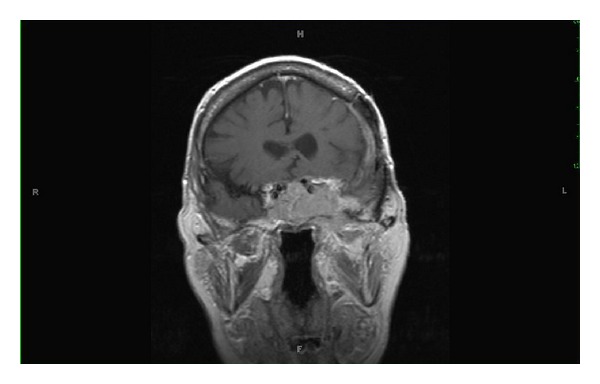# An Aggressive Sphenoid Wing Meningioma Causing Foster Kennedy Syndrome

**DOI:** 10.1155/2012/102365

**Published:** 2012-05-17

**Authors:** Harpreet S. Walia, F. Lawson Grumbine, Gagan K. Sawhney, David S. Risner, Neal V. Palejwala, Matthew E. Emanuel, Sandeep S. Walia

**Affiliations:** ^1^School of Medicine, Emory University, Atlanta, GA, USA; ^2^School of Medicine, Texas Tech University, Lubbock, TX, USA

## Abstract

Foster Kennedy syndrome is a rare neurological condition with ophthalmic significance that can manifest as acute visual loss. It is classically characterised by unilateral optic nerve atrophy and contralateral papilledema resulting from an intracranial neoplasm. Physicians should consider Foster Kennedy syndrome in patients who present with visual loss and who have a history of intracranial neoplasm. In addition to ophthalmologic examination, neuroimaging is essential for the diagnosis of Foster Kennedy syndrome.

## 1. Introduction

Foster Kennedy syndrome is a rare condition that classically involves optic nerve atrophy ipsilateral to an intracranial neoplasm with concomitant contralateral papilledema. As few as 37 cases have been completely documented between 1909 and 1989 [1]. We review the pathogenesis and common clinical manifestations of Foster Kennedy syndrome and highlight the role of neuroimaging in diagnosis.

## 2. Case History

A 72-year-old white female with a medical history of an aggressive left sphenoid wing meningioma, initially treated with resection and subsequently with radiotherapy for a recurrence four years, presented complaining of acute visual loss in her left eye. She also endorsed an associated left-sided retroorbital headache; she denied nausea, vomiting, or gait abnormalities. 

On physical exam, the patient had stable vital signs and was in no acute distress. Ophthalmologic exam revealed visual acuity of 20/50 in the right eye and finger counting in the left eye. A relative afferent pupillary defect was present in the left eye. Confrontation visual fields were full in the right eye but revealed significant generalized constriction in all four quadrants in the left eye. Slit lamp exam was remarkable for only moderate nuclear sclerotic cataracts in both eyes. On funduscopic exam, the right eye revealed a hyperemic, edematous optic disc with tortuous and dilated vessels and scattered drusen in the macula without subretinal fluid, and the left eye revealed a pale optic disc with scattered drusen in the macula without subretinal fluid. 

**Figure 1:**
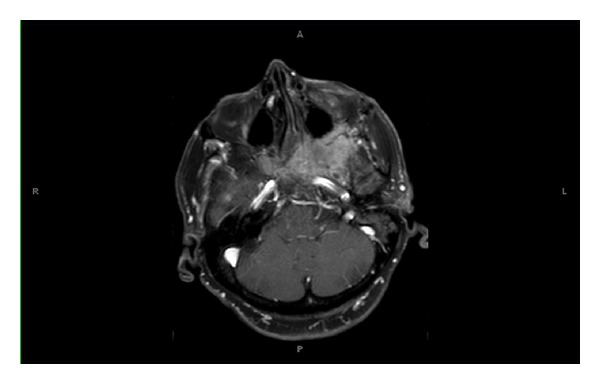


**Figure 2:**
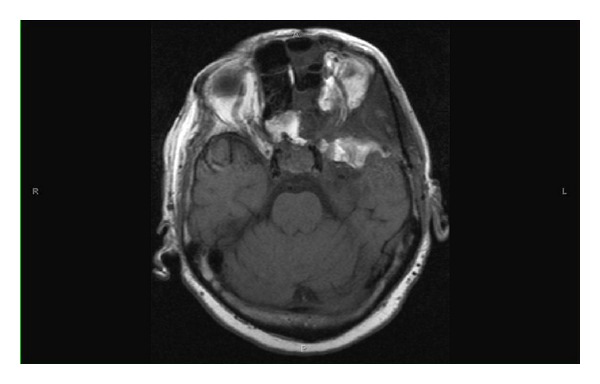


Given her history of known sphenoid wing meningioma, neuroimaging with MRI was obtained. Axial MRI scans revealed a large 4.4 cm × 4 cm × 3.4 cm mass in the left cavernous sinus extending into the left optic nerve and optic chiasm (see Figures 1 and 2). A coronal MRI scan confirmed infiltration into the sella turcica and sphenoid sinus (Figure 3). A lumbar puncture confirmed elevated intracranial pressure at 22 mmHg and did not show any signs of infection. Other diagnostic tests including complete blood count, complete metabolic panel, erythrocyte sedimentation rate, c-reactive protein, and electrocardiogram were unremarkable. Neuroimaging evidence of a cavernous sinus mass in the clinical scenario of a patient presenting with recurrent sphenoid wing meningioma with ipsilateral optic disc pallor and contralateral optic disc edema, along with elevated intracranial pressure, confirmed the Foster Kennedy syndrome.

## 3. Discussion

Foster Kennedy syndrome is a rare entity found with intracranial neoplasms. First described in 1911, the Foster Kennedy syndrome (also known as Gowers-Paton-Kennedy syndrome) [2] originates from a retrobulbar compressive optic neuropathy commonly caused by sphenoid wing meningioma, frontal lobe glioma, optic neuroglioma, olfactory glioma, chiasmal glioma, and craniopharyngioma [3]. Although more commonly associated with neoplasm, the syndrome can also be caused by vascular lesions, meningitis, internal carotid artery sclerosis, and Paget's disease of the skull [3, 4]. 

The pathogenesis for the clinical findings cannot be attributed to a single mechanism. It is postulated that initially a pressure, often secondary to intracranial mass, arises on one side of the optic nerve. The increased cerebrospinal fluid pressure causes impaired ocular venous return and compression in the subarachnoid space of the intraorbital part of the optic nerve [5]. This mechanical compression results in atrophy of the ipsilateral nerve fiber layer, which in turn prevents the development of papilledema [5]. Concurrently, the pressure is transmitted to the contralateral nerve so that it is under a relatively higher pressure, but without mechanical compression. This elevated pressure without compression results in papilledema [4]. In the early stages of the syndrome, the contralateral papilledema often precedes the ipsilateral optic atrophy, and visual acuity can be retained with only mild pallor on funduscopic examination [6].

Anosmia and headache are often present in true Foster Kennedy syndrome [4]; however, they are not universally present signs. Other common complications depend on the area of intracranial involvement and potentially include emotional lability, memory loss, nausea, vomiting, vertigo, hearing loss, extremity weakness, and facial paresis. Various ophthalmic signs and symptoms can be present depending on the localization of the tumor. Visual loss has been reported to be present in 70% of cases, visual field defects, most notably central scotomata, in 9%, and extraocular motility limitation in 6% [5]. Transient visual obscurations can be present due to fluctuations in intracranial or systemic blood pressure that cause transient compromise to the optic nerve [7]. 

Foster Kennedy syndrome is a rare constellation of clinical symptoms and signs that may present emergently with visual loss or decreased visual acuity. Neuroimaging, as warranted by clinical suspicion from history of ocular complaints and ophthalmologic exam findings, can assist physicians in determining the cause, such as intracranial neoplasm, of Foster Kennedy syndrome. Physicians should consider Foster Kennedy syndrome in patients who present acutely with visual loss and who have a history of intracranial neoplasm. In addition to ophthalmologic examination, neuroimaging to evaluate for intracranial neoplasm is essential for the diagnosis of Foster Kennedy syndrome. Treatment options are aimed at resolving the underlying cause of the syndrome and are beyond the scope of this case report.

## Figures and Tables

**Figure 3:**